# Co-Targeting PIM Kinase and PI3K/mTOR in NSCLC

**DOI:** 10.3390/cancers13092139

**Published:** 2021-04-29

**Authors:** Gillian Moore, Clara Lightner, Samira Elbai, Lauren Brady, Siobhan Nicholson, Ronan Ryan, Katie E. O’Sullivan, Kenneth J. O’Byrne, Carmen Blanco-Aparicio, Sinead Cuffe, Michael O’Neill, Susan Heavey, Stephen P. Finn, Kathy Gately

**Affiliations:** 1 Department of Clinical Medicine, Trinity Translational Medicine Institute, St. James’s Hospital, Dublin, Ireland; gillianmoore@rcsi.ie (G.M.); lightnec@tcd.ie (C.L.); elbais@tcd.ie (S.E.); scuffe@stjames.ie (S.C.); 2 School of Pharmacy and Biomolecular Sciences, RCSI, Dublin, Ireland; 3 Department of Histopathology, St. James’s Hospital, Dublin, Ireland; bradyl4@tcd.ie (L.B.); snicholson@stjames.ie (S.N.); stephen.finn@tcd.ie (S.P.F.); 4 Department of Cardio-Thoracic Surgery, St. James’s Hospital, Dublin, Ireland; rryan@stjames.ie (R.R.); kaosulli@me.com (K.E.O.); 5 Princess Alexandra Hospital, Translational Research Institute and The School of Biomedical Sciences, Institute of Health and Biomedical Innovation, Queensland University of Technology, Brisbane, QLD 4102, Australia; Kenneth_OByrne@health.qld.gov.au; 6 Experimental Therapeutics Program, Spanish National Cancer Research Centre (CNIO), C/Melchor Fernández Almagro 3, 28029 Madrid, Spain; cblanco@cnio.es; 7 Inflection Biosciences Ltd., Blackrock Co., Dublin, Ireland; moneill@inflectionbio.com; 8 Division of Surgery and Interventional Science, University College London, London WC1E 6BT, UK; s.heavey@ucl.ac.uk

**Keywords:** PIM kinase, PI3K-mTOR, c-Myc, drug resistance, NSCLC, tumor tissue explants, miRNA, biomarker

## Abstract

**Simple Summary:**

PIM kinases interact with major oncogenic players, including the PI3K/Akt pathway, and provide an escape mechanism leading to drug resistance. This study examined PIM kinase expression in NSCLC and the potential of PIM1 as a prognostic marker. The effect on cell signaling of novel preclinical PI3K/mTOR/PIM kinase inhibitor IBL-301 was compared to PI3K/mTOR inhibition in vitro and ex vivo. PI3K-mTOR inhibitor sensitive (H1975P) and resistant (H1975GR) cells were compared for altered IL6/STAT3 pathway expression and sensitivity to IBL-301. All three PIM kinases are expressed in NSCLC and PIM1 is a marker of poor prognosis. IBL-301 inhibited c-Myc, the PI3K-Akt and JAK/STAT pathways in vitro and in NSCLC tumor tissue explants. IBL-301 also inhibited secreted pro-inflammatory cytokine MCP-1. PIM kinases were activated in H1975GR cells which were more sensitive to IBL-301 than H1975P cells. A miRNA signature of PI3K-mTOR resistance was validated. Co-targeting PIM kinase and PI3K-mTOR warrants further clinical investigation.

**Abstract:**

PIM kinases are constitutively active proto-oncogenic serine/threonine kinases that play a role in cell cycle progression, metabolism, inflammation and drug resistance. PIM kinases interact with and stabilize p53, c-Myc and parallel signaling pathway PI3K/Akt. This study evaluated PIM kinase expression in NSCLC and in response to PI3K/mTOR inhibition. It investigated a novel preclinical PI3K/mTOR/PIM inhibitor (IBL-301) in vitro and in patient-derived NSCLC tumor tissues. Western blot analysis confirmed PIM1, PIM2 and PIM3 are expressed in NSCLC cell lines and PIM1 is a marker of poor prognosis in patients with NSCLC. IBL-301 decreased PIM1, c-Myc, pBAD and p4EBP1 (Thr37/46) and peIF4B (S406) protein levels in-vitro and MAP kinase, PI3K-Akt and JAK/STAT pathways in tumor tissue explants. IBL-301 significantly decreased secreted pro-inflammatory cytokine MCP-1. Altered mRNA expression, including activated PIM kinase and c-Myc, was identified in Apitolisib resistant cells (H1975GR) by an IL-6/STAT3 pathway array and validated by Western blot. H1975GR cells were more sensitive to IBL-301 than parent cells. A miRNA array identified a dysregulated miRNA signature of PI3K/mTOR drug resistance consisting of regulators of PIM kinase and c-Myc (miR17-5p, miR19b-3p, miR20a-5p, miR15b-5p, miR203a, miR-206). Our data provides a rationale for co-targeting PIM kinase and PI3K-mTOR to improve therapeutic response in NSCLC.

## 1. Introduction

The pro-viral insertion site in Moloney murine leukemia virus (PIM) proteins are evolutionarily conserved serine/threonine kinases in eukaryotes consisting of PIM1, PIM2 and PIM3. PIM kinases regulate cell survival pathways, are implicated in the progression of lung cancer [1,2] and play a role in enhanced resistance to chemotherapy [3,4] and molecular targeted therapies of EGFR, MET and PI3K/Akt/mTOR signaling [5,6,7]. The tumorigenic potential of the PIM family is mainly mediated by its interactions with other pathways commonly upregulated in cancer such as the PI3K (Phosphoinositide 3-kinase) pathway. Both PIM and PI3K phosphorylate overlapping substrates to activate common pathways that control various physiological processes that ultimately dictate the balance between cell survival and apoptosis. PIM1 enhances the transforming potential of oncogenes such as c-Myc [8] and has been shown to promote EMT and stemness in IL-6-induced breast cancer cells via c-Myc activation [9]. Cen et al. showed that inhibition of Akt led to transcriptional induction of PIM1 kinase which in turn regulated the expression of RTKs in prostate cancer [5]. We have shown that a co-targeted approach is more effective compared to single PIM kinase or PI3K-mTOR inhibition, as previously reported in prostate cancer [10].

The PI3K/Akt/mTOR signaling pathway is activated in 50–70% of NSCLC [11] and plays an overarching role in all the hallmarks of cancer including evading immune destruction. We previously developed NSCLC cell line models of acquired resistance to PI3K-mTOR inhibitor Apitolisib to pinpoint specific bypass mechanisms of resistance [12]. These resistant cell lines displayed an epithelial to mesenchymal transition (EMT) phenotype and also demonstrated resistance to another PI3K/mTOR inhibitor (Dactolisib (BEZ235)) highlighting their use as a universal cell line model of resistance to PI3K-mTOR inhibition [12]. In lung cancer cell lines, pro-inflammatory cytokine IL-6 and chemokine MCP-1 synergistically induce EMT in a twist/STAT3-dependent fashion [13]. Previous studies have highlighted activation of the IL-6 cytokine pathway and STAT3, along with EMT features in NSCLC cells with acquired resistance to molecularly targeted drugs including PI3K inhibition [14].

This study evaluated PIM kinase expression in NSCLC and in response to PI3K-mTOR inhibition. We showed PIM1, PIM2 and PIM3 are expressed in NSCLC cells and highlighted the potential of elevated PIM1 expression as a prognostic marker in patients with NSCLC. We investigated the efficacy and effect on cell signaling of novel preclinical PI3K/mTOR/PIM kinase inhibitor (IBL-301) in NSCLC cell lines and patient tumor tissue explants. NSCLC cell lines were sensitive to IBL-301 in the nanomolar range and it was effective at inhibiting co-regulator c-Myc. It also showed promising inhibition of MAP kinase, PI3K-Akt and JAK/STAT pathways in NSCLC tumor tissue explants and decreased secreted MCP-1 in conditioned media from both tissue explants and resistant cell lines. PIM kinase and c-Myc were activated in PI3K-mTOR inhibitor resistant cells which were more sensitive to IBL-301 than parent cells. A miRNA signature of PI3K/mTOR drug resistance was validated consisting of regulators of PIM kinase and c-Myc (miR17-5p, miR19b-3p, miR20a-5p, miR15b-5p, miR203a, miR-206). Taken together, these findings indicate that co-targeting both PIM kinase and PI3K-mTOR pathways has the potential to be more efficacious and may provide a more durable response to treatment in patients with an activated PIM kinase and/or PI3K-mTOR pathway.

## 2. Materials and Methods

### 2.1. Cell Lines and Drugs

NSCLC cell lines HCC827, H460, H1975 and Calu-6 were purchased from the European Collection of Authenticated Cell Cultures (ECACC). Apitolisib (GDC-0980), a PI3K/mTOR inhibitor, Dactolisib (BEZ235), a PI3K/mTOR inhibitor, pan-PI3K inhibitor (GDC-0941) and pan-PIM kinase inhibitor (AZD1208) were purchased from Selleck Chemicals. IBL-301, a triple targeting PI3K/mTOR/PIM inhibitor (licensed by Inflection Biosciences from CNIO) was provided by Inflection Biosciences Ltd. (Figure S1). GDC-0980 resistant H1975 cells (H1975GR) were developed as described previously (19). H1975GR and age-matched parent control cells (H1975P) were grown in RPMI1640 media (Lonza) supplemented with 10% FBS and 1% penicillin/streptomycin at 37 °C and 5% CO2. H1975GR were maintained in 0.58 µM GDC-0980.

### 2.2. Patient Samples for Immunohistochemistry

Following ethical approval, a tissue microarray (TMA) containing quadruplicate cores of 134 NSCLC patients (56 adenocarcinomas, 65 squamous cell carcinomas and 13 of other histology) of various stages from St James’s Hospital, Dublin, Ireland was used for immunohistochemical analysis. Patient clinicopathological characteristics are shown in Table S1.

### 2.3. Immunohistochemical Detection Methods

Immunohistochemistry was performed on 5 μm TMA sections. Slides were deparaffinized, rehydrated, washed and quenched according to standard protocol. For antigen retrieval, slides were incubated in Tris/EDTA, pH 9.0, for 15 min at 105 °C. For PIM1 antibody staining, slides were incubated with rabbit monoclonal (EP2645Y) (Abcam Cat# ab75776) primary antibody diluted in PBS (1:100) and 2.5% normal serum for 60 min at ambient temperature (VECTASTAIN**^®^** Elite**^®^** ABC Kit, Vector Laboratories, Oxfordshire, UK). Overall staining intensity of tumor tissue (i.e., both nuclear and cytoplasmic) was evaluated by pathologists (S.F & C.L) and assessed using the Allred scoring system, where a positive score was 3.0 or higher only.

### 2.4. Western Blotting

Anti-PIM-1 (Cat# 3247) or anti-PIM-1 (Cat#2907), anti-PIM-2 (Cat# 4730), anti-PIM-3 (Cat# 4165), anti-c-Myc (Cat# 9402), anti-p21 (Cat# 2947), anti-mTOR (Cat# 2983) and anti-phospho-mTOR (Ser2448) (Cat# 2971), anti-pAKT (Ser473) (Cat# 4058), anti-4EBP1 (Cat# 9644), anti-phospho-S6 (Ser240/244) (Cat# 2215), anti-phospho-4EBP1 (Thr37/46) (Cat# 2855), anti-phospho-eIF4B (Ser406) (Cat# 5399) and anti-αβ-tubulin (Cat #2148) rabbit primary antibodies were purchased from Cell Signaling Technology and diluted as per manufacturer’s protocol. Separation of 50 µg or 30 µg of protein was done with a Western blot and carried out as previously described [12].

### 2.5. Cell Proliferation and Cell Viability Assays

Cell proliferation of H1975P and H1975GR was measured using a BrdU assay (Roche Diagnostics Ltd.). Cell viability was measured by the CellTiter-Glo**^®^** assay (Promega), respectively. Cells were seeded at 2 × 10^3^ cells overnight into adherent 96-well plates in complete cell culture medium. Cells were treated with IBL-301 for 72 h at a range of concentrations (0.0195 μM–1.25 μM) as described in triplicate wells. Absorbance was measured at 450 nm, reference wavelength 690 nm. Following treatment with GDC-0941, AZD1208, or IBL-301 a volume of CellTiter-Glo**^®^** reagent equal to the volume of cell culture medium present in each well (100 μL) was added, contents were mixed for 2 min on an orbital shaker to induce cell lysis and the plate was incubated at room temperature for 10 min to stabilize luminescent signal.

### 2.6. Ex Vivo Cultured Lung Tumor Explants

Following ethical approval (joint St James’s Hospital/AMNCH Ethical Review Board) and written informed consent from nine patients prior to surgery, lung tumor tissue biopsies were obtained from resected tissue by a pathologist (Table S2). Fresh biopsies were cut into several equally sized pieces (approximately 1mm^3^) where possible. An individual piece of tumor was added to each well of a 12-well culture plate containing 1 mL of RPMI1640 media (Lonza) supplemented with 10% FBS and 1% penicillin/streptomycin. One of the following treatments were added to each well: 1% DMSO (vehicle control), 250 nM BEZ235 or 250 nM IBL-301. The plate was sealed with parafilm and incubated for 72 h at 37 °C and 5% CO_2_. Following 72 h culture, tumor conditioned media (TCM) was collected and stored at −80 °C. Vehicle control- and drug-treated tumor pieces were transferred to Allprotect**^®^** (Qiagen, Hilden, Germany), removed, snap frozen and stored at −80 °C.

### 2.7. Phospho-Protein Profiling

PathScan**^®^** intracellular signaling arrays (Cell Signaling Technologies, Danvers, MA, USA) were used to profile expression of 18 well characterized signaling molecules when phosphorylated or cleaved. Snap frozen lung tumor tissue and H1975P and H1975GR cell line pellets were homogenized and lysed as per protocol. All samples were diluted to 1.0 mg/mL and 75 µL of cell lysates were added per well and incubated for overnight at 4 °C on a shaker. Following manufacturer’s protocol, the slides were incubated in LumiGlo**^®^**/peroxide mix (chemiluminescent reagent) for the duration of slide imaging using a Fusion FX imaging platform (Vilber Lourmat, Collégien, France). Densitometry analysis was carried out using ImageJ https://imagej.nih.gov/ij/, accessed on: 20 April 2020.

### 2.8. IL6/STAT3 Signaling Gene Array

RNA was isolated from H1975P and H1975GR cells using the RNeasy mini kit (Qiagen, Hilden, Germany). cDNA was synthesized from 0.5 µg of total RNA and analysed for expression of a panel of 84 genes. Controls and house-keeping genes were quantified using the Human IL-6/STAT3 signaling RT2 Profiler PCR array (Qiagen). Changes in gene expression between parent H1975P and resistant H1975GR cells (*n* = 1) were analyzed using SABiosciences online software, incorporating the ΔΔCT method, and presented as a clustergram (Figure S2). 

### 2.9. Real Time PCR Analysis

Quantitative real-time PCR (qRT-PCR) was used to measure mRNA expression levels of MYC, PIM1, MTOR, SOCS1, CDKN1A1, BCL2, TNF and CD40 to validate the initial IL-6/STAT3 array data. The mRNA levels were quantified using gene specific primers (see Table S3 for primer sequences) (Integrated DNA Technologies Ltd., Coralville, IA, USA) and SYBR Green PCR Master Mix (Applied Biosystems, Foster City, CA, USA). The mean expression of the endogenous control genes B2M and RPLP0 were used to normalize all Ct values.

### 2.10. miRNA Expression Profiling

Custom 96-well miRNA PCR arrays were designed containing 19 miRNAs of interest, 3 endogenous controls (miR-103a-3p, miR-423-5p and miR-191-5p), and 2 positive controls for cDNA synthesis and PCR (UniSp3 and UniSp6, Table S4). RNA was isolated from H1975P and H1975GR (*n* = 3) using the miRCURY™ RNA isolation kit (Exiqon A/S, Vedbaek, Denmark). First-strand cDNA synthesis and real-time PCR amplification was carried out as per the miRCURY LNA™ Universal RT microRNA PCR protocol (Exiqon A/S). Each assay was run in duplicate wells on an ABI QuantStudio 5 (Applied Biosystems, Foster City, CA, USA). Results were pre-processed and analyzed using the downloadable Exiqon qPCR data analysis software, GenEx.

### 2.11. MILLIPLEX MAP Human Cytokine/Chemokine Magnetic Bead 10-Plex Panel

Eight cytokines and two chemokines were measured by multiplexed fluorescent bead-based immunoassay detection (MILLIPLEX MAP system, MerckMillipore, Burlington, MA, USA) according to the manufacturer’s instructions, using a combination of 10-plex (HCYTOMAG-60K-10). The 10-plex kit contained antibody-conjugated beads for the following cytokines and chemokines: MCP-1/CCL2, Rantes/CCL5, IFNγ, TNFα, IL-12(p70), IL-10, IL-1a, IL-1b, IL-6 and IL-8. Supernatant collected from 70% confluent H1975P and H1975GR cells, cultured under normal maintenance conditions and NSCLC patient-derived TCM (as described previously) were assayed undiluted with the 10-plex. For each assay, the curve was derived from various concentrations of the cytokine/chemokine standards assayed in the same manner as patient and cell line samples.

### 2.12. In-Silico Data Analysis

In-silico data analysis was performed using the lung cancer specific Kaplan–Meier plotter (http://kmplot.com/analysis/index.php?p=service&cancer=lung, accessed on: 20 April 2020). The system includes gene chip and RNA-seq data from GEO, EGA, and TCGA sources. The primary purpose of the tool is a meta-analysis based discovery and validation of survival biomarkers [15].

### 2.13. Statistical Analysis

All statistical analysis was carried out using GraphPad Prism software (RRID:SCR_002798) version 5.01 (GraphPad software Inc., San Diego, CA, USA). In the case where two groups were compared, a paired student t-test was used. In the case of of MCP-1 levels in tumor explants following drug treatment, a one-way ANOVA with Dunnett’s multiple comparisons test was use. In the case of the PathScan array data from tumor explants, the grouped data was analyzed by a two-way ANOVA with Dunnett’s multiple comparisons test. All *p*-values reported are two tailed and * *p* (<0.05), ** *p* (<0.01), *** *p* (<0.001).

## 3. Results

### 3.1. PIM1, PIM2 and PIM3 Expression in HCC827, H460 and H1975 Cell Lines and Investigating PIM1 Expression as a Prognostic Marker in NSCLC

**Figure 1:**
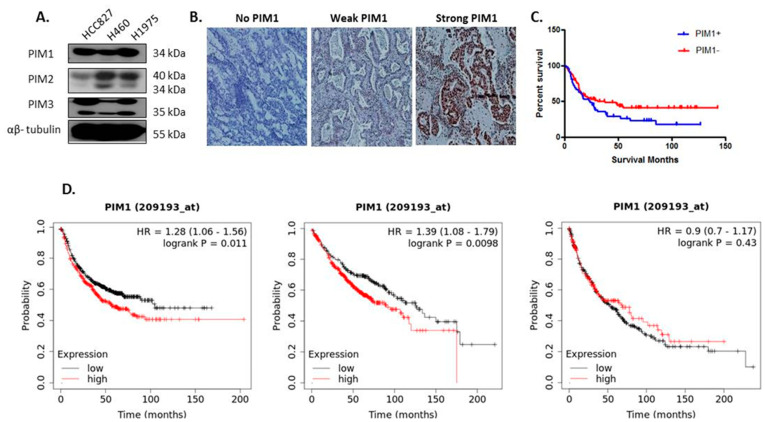
PIM1, PIM2 and PIM3 expression in NSCLC cell lines and PIM1 biomarker potential in NSCLC patients. (**A**) Representative Western blot images showing PIM1, PIM2 and PIM3 expression in HCC827, H460 and H1975 cell lines. Original Western blots in Figure S3. (**B**) Representative images of PIM1 staining. No expression, weak nuclear and strong nuclear and moderate cytoplasmic PIM1 positivity in NSCLC tissue from a Grade 2 tumor is shown at low (×10) magnification. (**C**) Overall PIM1 positive patients (i.e., those with an Allred score of ≥3) had a median survival time of 23.67 months in comparison to 37.32 months in the PIM1 negative group (*n* = 134, Log-rank test *p* = 0.056). (**D**) KM Plotter survival analysis of PIM1 mRNA levels in lung squamous and adenocarcinoma patients (*n* = 982) is associated with reduced progression free survival time (HR = 1.28, *p* < 0.05). A sub-analysis of adenocarcinoma cases only (*n* = 719), PIM1 mRNA expression is associated with poorer overall survival (HR = 1.39, *p* < 0.01). There was no association between PIM1 mRNA expression and overall survival in a sub-analysis of squamous carcinoma cases (*n* = 524, HR = 0.9, *p* = 0.43). Source: www.kmplot.com (accessed on 20 April 2020).

PIM1, PIM2 and PIM3 proteins were expressed in HCC827, H460 and H1975 cell lines as determined by Western blot analysis. The anti-PIM1 (C93F2) antibody was used which detects the smaller 34 kDa (PIM1S) protein only and all three cell lines expressed the 34 kDa PIM1S protein. The anti-PIM2 (D1D2) antibody was used which detects all three isoforms (34, 38 and 40 kDa) of PIM2. The H460 cell line had high expression of both the 34 kDa and 40 kDa PIM2 isoforms while HCC827 cells had weak expression of both PIM2 isoforms and H1975 cells had very weak expression of the 34 kDa isoform and high expression of the 40 kDa isoform. The anti-PIM3 (D17C9) antibody was used which detects a 35 kDa protein. All three cell lines expressed the 35 kDa protein (Figure 1A and Figure S3).

PIM1 expression was examined in 134 NSCLC patients (56 adenocarcinomas, 65 squamous cell carcinomas and 13 of other histology) of various stages (I–IV) by immunohistochemical analysis (Table S1). Representative images of PIM1 positivity in NSCLC tissue is shown in Figure 1B. Overall PIM1 positive patients (i.e., those with an Allred score of ≥3) had a median survival time of 23.67 months in comparison to 37.32 months in the PIM1 negative group (*n* = 134, Log-rank test *p* = 0.056) (Figure 1C). There was no correlation with PIM1 expression and tumor grade. A publicly available Kaplan–Meier analysis of fourteen transcriptomic databases including TCGA indicates a significant association between PIM1 mRNA levels in lung squamous/adenocarcinoma patients and reduced progression free survival (Figure 1D, *n* = 982 HR = 1.28, *p* = 0.011) [15]. A sub-analysis of lung adenocarcinoma cases only indicates a significant association between PIM1 mRNA expression and reduced overall survival (Figure 1D, *n* = 719 HR = 1.39, *p* = 0.0098).

### 3.2. IBL-301

#### 3.2.1. Comparing the Efficacy of pan-PI3K Inhibitor (GDC-0941), pan-PIM Kinase Inhibitor (AZD1208), to Triple Targeted PI3K/mTOR/PIM Inhibitor (IBL-301)

**Figure 2:**
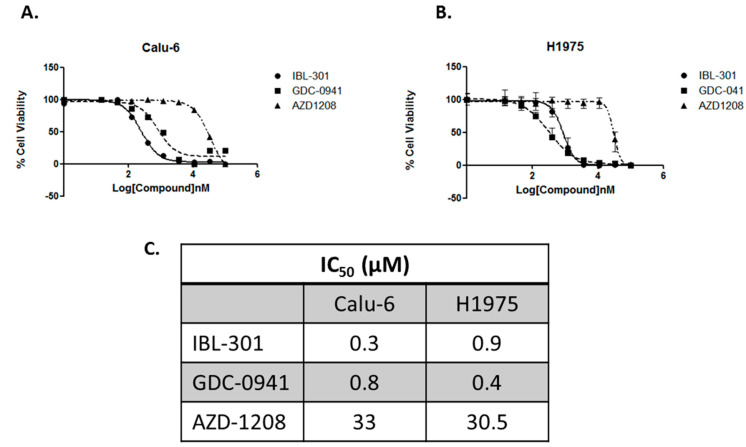
Dose response curves of IBL-301, GDC-0941 and AZD1208 in Calu-6 and H1975 cell lines. The dose response effect of triple targeted PI3K/mTOR/PIM inhibitor (IBL-301), pan-PI3K inhibitor (GDC-0941) and pan-PIM kinase inhibitor (AZD1208) (100 μM, 33.33 μM, 11.11 μM, 3.70 μM, 1.23 μM, 0.41 μM, 0.13 μM, 0.04 μM, 0.01 μM and DMSO vehicle) on cell viability over 72 h was measured by CellTiter-Glo^®^ assay in (**A**) Calu-6 and (**B**) H1975 cell lines. Values are reported as mean ± SEM (*n* = 2). (**C**) IC50 values were calculated representing the percentage of inhibition against compound concentration and adjusting the experimental data to sigmoidal curve using the software GraphPad Prism 5.01.

To investigate the efficacy of novel first-in-class triple targeted PI3K/mTOR/PIM inhibitor (IBL-301) (Inflection Biosciences Ltd., Dublin, Ireland) in comparison to single targeted agents pan-PI3K inhibitor (GDC-0941) and pan-PIM kinase inhibitor (AZD1208), IC50 values were determined via the CellTiter-Glo**^®^** cell viability assay in Calu-6 and H1975 NSCLC cell lines (Figure 2A,B). Both cell lines were more sensitive to IBL-301 (0.3 μM and 0.9 μM respectively) and GDC-0941 (0.8 μM and 0.4 μM respectively) than AZD1208 (33 μM and 30.5 μM respectively) (Figure 2C).

#### 3.2.2. The Effect of Triple Targeted Inhibitor IBL-301 on Intracellular Signaling Pathways In-Vitro

**Figure 3:**
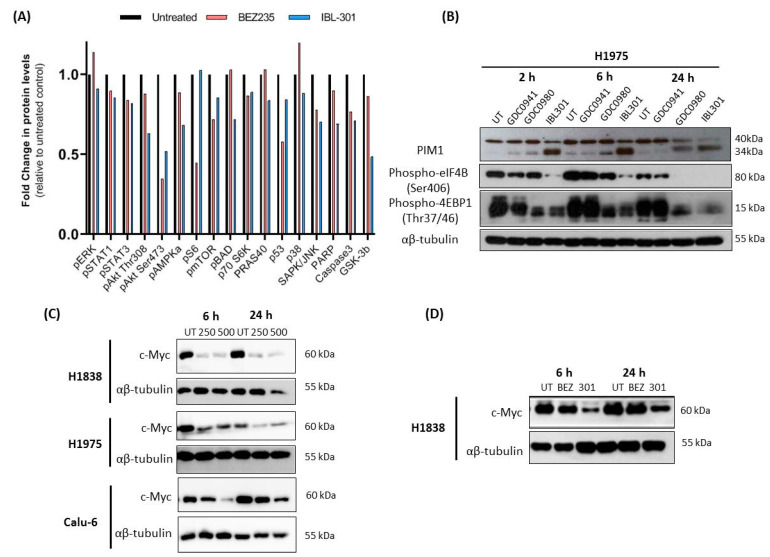
IBL-301 alters NSCLC cell line protein expression and signaling pathways. (**A**) H1975 cells were treated with DMSO vehicle control, 250 nM PI3K/mTOR inhibitor BEZ235 or 250 nM PI3K/mTOR/PIM inhibitor IBL-301 for 24 h, protein was extracted and assayed using a PathScan^®^ intracellular signaling array (*n* = 1). The top altered pathways by IBL-301 were Akt (Thr308 and Ser473), AMPKa, BAD, GSK-3B and SAPK/JNK while the top altered pathways by BEZ235 were Akt (Ser473), S6, mTOR and p53. (**B**) H1975 cells were treated with DMSO vehicle control (UT), 250 nM of PI3K inhibitor GDC0941, PI3K/mTOR inhibitor GDC0980 or PI3K/mTOR/PIM inhibitor IBL-301 for 2, 6 and 24 h, followed by protein extraction and Western blotting for PIM1, p-4EBP1 (Thr37/46), p-eIF4B (Ser406) and endogenous control αβ-tubulin. After an initial increase in PIM-1S across all treatments, expression decreased at 24 h. Across all time points, IBL-301 was most effective at inhibiting PIM-1L expression and phosphorylation of 4EBP1 (Thr37/46) and eIF4B (Ser406). (**C**) NSCLC cell lines H1838, H1975 and Calu-6 were treated with 250 nM and 500 nM IBL-301 for 6 and 24 h followed by protein extraction and Western blotting for c-Myc and αβ-tubulin. Both drug concentrations decreased c-Myc expression across the time points in the H1838 and the H1975 cells, while only 500 nM IBL-301 decreased c-Myc in Calu-6 at 6 and 24 h. (**D**) H1838 were treated with DMSO control (UT), 250 nM BEZ235 or 250 nM IBL-301 for 6 and 24 h, followed by protein extraction and Western blotting for c-Myc and αβ-tubulin. Using 250 nM IBL-301 reduced c-Myc levels at both time points while in contrast 250 nM BEZ235 had no observed effect on c-Myc across the time points tested. Original Western blots for Figure 3B–D in Figures S4–S6.

H1975 cells were treated with DMSO vehicle control (untreated: UT), 250 nM PI3K/mTOR inhibitor Dactolisib/BEZ235 or 250 nM PIM/PI3K/mTOR inhibitor IBL-301 for 24 h and extracted proteins were assayed using a PathScan^®^ intracellular signaling array (*n* = 1). The most altered phosphorylated proteins by IBL-301 were Akt (Thr308 and Ser473), AMPKa, BAD, SAPK/JNK and GSK-3b while the top altered phosphorylated proteins by BEZ235 were Akt (Ser473), S6, MTOR and p53 (Figure 3A).

H1975 cells were treated with DMSO vehicle control (UT), 250 nM of PI3K inhibitor GDC0941, PI3K/mTOR inhibitor GDC0980 or PIM/PI3K/mTOR inhibitor IBL-301 for 2, 6 and 24 h. Expression of both PIM1S (34 kDa) and PIM1L (44 kDa) were examined. Across all time points, IBL-301 was most effective at inhibiting PIM1L protein expression. Interestingly, all inhibitors resulted in an initial increase in PIM1S at 2 and 6 h time-points with IBL-301 resulting in the most significant increase. This increase in PIM1S was most effectively inhibited by IBL-301 at 24 h. IBL-301 was most effective at inhibiting phosphorylation of 4EBP1 (Thr37/46) and eIF4B (Ser406) (Figure 3B). The three NSCLC cell lines H1838, H1975 and Calu-6 were treated with 250 nM and 500 nM IBL-301 for 6 and 24 h. Both IBL-301 concentrations decreased c-Myc expression across all time points in the H1838 cells and the H1975 cells, while only 500 nM IBL-301 decreased c-Myc in Calu-6 cells at the 6 and 24 h time point (Figure 3C). H1838 cells were treated with DMSO control (UT), 250 nM BEZ235 or 250 nM IBL-301 for 6 and 24 h. As previously shown, 250 nM IBL-301 reduced c-Myc levels at both time points while in contrast, 250 nM BEZ235 had no observed effect on c-Myc levels at the time points tested (Figure 3D).

#### 3.2.3. The Effect of Triple Targeted Inhibitor IBL-301 on Intracellular Signaling Pathways in Cultured NSCLC Tumor Explants

**Figure 4:**
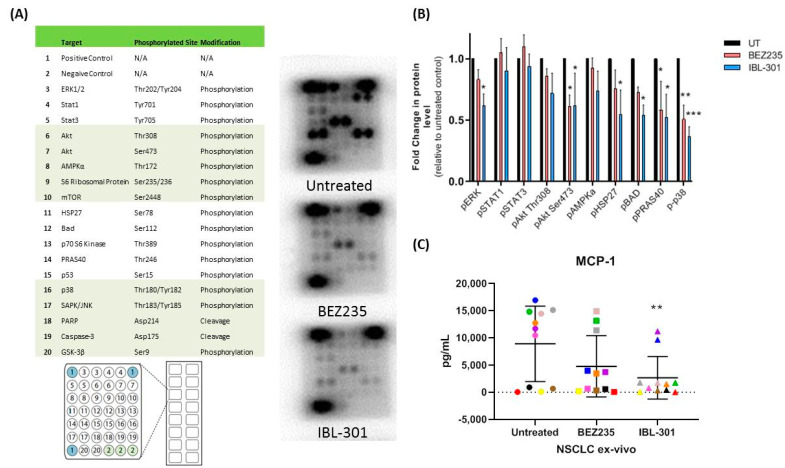
Targeting PI3K/mTOR or PI3K/mTOR/PIM in NSCLC explant tissue. (**A**) NSCLC tumor explants were cultured ex vivo for 72 h with DMSO vehicle control, 250 nM BEZ235 or 250 nM IBL-301 and protein was extracted and assayed using a PathScan^®^ intracellular signaling array (*n* = 3). (**B**) Ten out of eighteen proteins as indicated were detectable, IBL-301 was more effective at altering the phosphorylation profile of the NSCLC tumor than BEZ235. IBL-301 significantly decreased pERK, pAkt (Ser473), pHSP27, pPRAS40 and p-p38 while BEZ235 significantly decreased pAkt (Ser473), pPRAS40 and p-p38 (two-way ANOVA with Dunnett’s multiple comparisons test was used, * *p* < 0.05, ** *p* < 0.01, *** *p* < 0.001). (**C**) Tissue conditioned media from cultured NSCLC explants were assayed for MCP-1 levels using a magnetic bead based immunoassay (MILLIPLEX MAP) (*n* = 11) and IBL-301 significantly decreased MCP-1 secretions (one-way ANOVA, *p* < 0.01).

NSCLC tumor explants (Table S2) were cultured ex-vivo for 72 h with DMSO vehicle control (UT), 250 nM BEZ235 or 250 nM IBL-301. Extracted proteins were analyzed using a PathScan^®^ intracellular signaling array (*n* = 3) (Figure 4A). Ten out of eighteen proteins were detectable and both IBL-301 and BEZ235 effectively altered the phosphorylation profile of NSCLC tumors with only IBL-301 significantly suppressing p-BAD expression as expected. IBL-301 also significantly decreased pERK, pAkt (Ser473), pHSP27, pPRAS40 and p-p38 while BEZ235 significantly decreased pAkt (Ser473), pPRAS40 and p-p38 (Figure 4B). Tissue conditioned medias from cultured NSCLC explants were assayed for MCP-1 levels using a magnetic bead based immunoassay (MILLIPLEX MAP) (*n* = 11 and IBL-301 significantly decreased MCP-1 secretions (one-way ANOVA, *p* < 0.01) (Figure 4C).

### 3.3. Acquired Resistance to PI3K/mTOR Inhibitors Alters the Protein Expression Profile of PIM1, and Gene Expression of IL-6/STAT3 Signaling Pathway Members Including PIM1 and MYC in the H1975GR Cells

**Figure 5:**
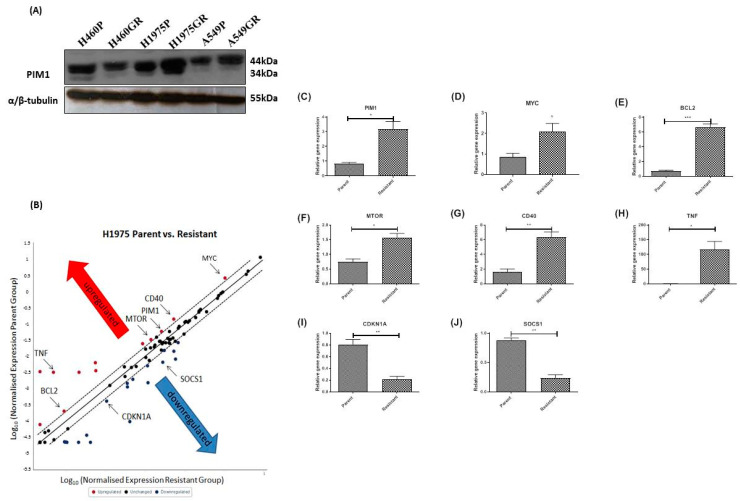
PIM1 expression in PI3K/mTOR inhibitor resistant cell line models and differential IL-6/STAT3 pathway signaling in H1975GR cells compared to H1975P cells. (**A**) Elevated PIM1 expression in H1975GR and A549GR cells by Western blot. Original Western blots in Figure S7. (**B**) The scatter plot compares normalized expression of every gene on the array between H1975GP and H1975GR. Twelve genes were upregulated (red dots) greater than two-fold and nineteen genes were downregulated (green dots) greater than two-fold in H1975GR. As indicated on the scatter plot, 8 genes altered by ≥2-fold in the array were chosen for further validation by SYBR-based real-time quantitative PCR. There was an upregulation of (**C**) PIM1 (3.84-fold, *p* < 0.05), (**D**) MYC (2.42-fold, *p* < 0.05), (**E**) anti-apoptotic BCL2 (9.23-fold, *p* < 0.001), (**F**) mTOR (2.10-fold, *p* < 0.05), (**G**) the TNF receptor co-stimulatory molecule CD40 (3.94-fold, *p* < 0.01) and (**H**) TNF (>100-fold, *p* < 0.05). In contrast there was a downregulation of (**I**) CDKN1A (−3.82-fold, *p* < 0.01) and (**J**) SOCS1 (−3.67-fold, *p* < 0.01). * *p* < 0.05, ** *p* < 0.01, *** *p* < 0.001, paired student *t*-test, *n* = 4.

PIM1L expression was activated in PI3K/mTOR inhibitor resistant H1975GR and A549GR cells compared to matched parent control cells and a decrease in PIM1L was shown in H460GR cells (Figure 5A). RNA from H1975GR and matched parent control cells were screened using the Human IL-6/STAT3 RT2 Profiler Array (Qiagen, Hilden, Germany). Using a standard two-fold increase or decrease in gene expression as the cut-off for gene candidate selection. Twelve genes were upregulated and nineteen genes were downregulated >2-fold in the H1975GR compared to the parent cell line (Figure 5B and Figure S2). *PIM1* and *MYC* were both overexpressed in H1975GR. *PIM1* and *MYC*, and a subset of upregulated genes (*BCL2*, *MTOR*, *TNF* and *CD40*) and downregulated genes (i.e., *CDKN1A1* and *SOCS1*) were further validated in independent replicates (n ≥ 3) using quantitative real-time PCR. There was a significant upregulation of *PIM1* (3.84-fold, *p* < 0.05) (Figure 5C), *MYC* (2.42-fold, *p* < 0.05) (Figure 5D), anti-apoptotic *BCL2* (9.23-fold, *p* < 0.001) (Figure 5E), *MTOR* (2.10-fold, *p* < 0.05) (Figure 5F), and the gene coding the TNF receptor co-stimulatory molecule *CD40* (3.94-fold, *p* < 0.01) (Figure 5G). While the *TNF* gene was relatively unexpressed (average Ct value > 33) in the parent cell line, it was markedly re-expressed (average Ct value = 26) in the H1975GR (>100-fold, *p* < 0.05) (Figure 5H). In contrast there was a significant downregulation of *CDKN1A* (−3.82-fold, *p* < 0.01) (Figure 5I) and *SOCS1* (−3.67-fold, *p* < 0.01) (Figure 5J).

### 3.4. Protein Evaluation of Identified Genes Associated with PI3K/mTOR Inhibitor Resistance in H1975GR Cells

**Figure 6:**
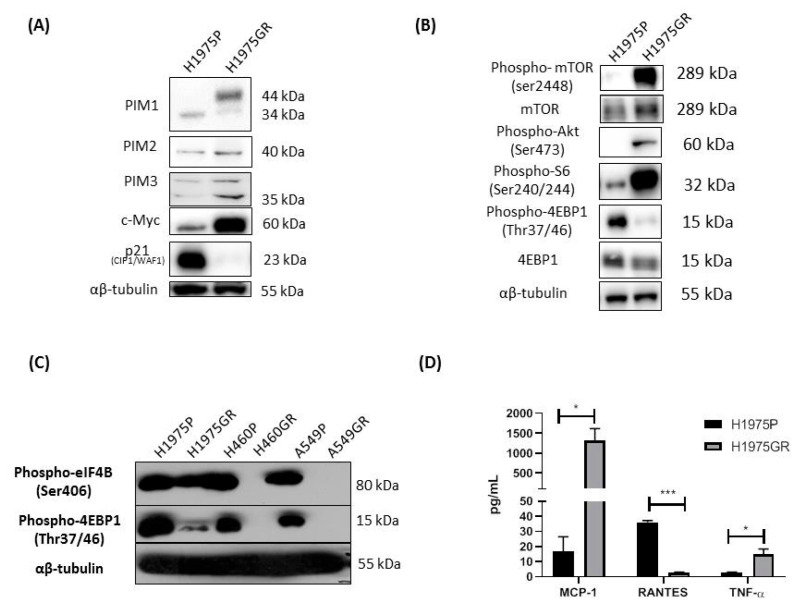
Protein evaluation of identified genes associated with PI3K/mTOR inhibitor resistance in the H1975GR cells. (**A**). Representative Western blot images showing significant upregulation of PIM1L (*p* < 0.01), PIM2 (*p* = 0.054), PIM3 (*p* < 0.05), c-Myc (*p* < 0.05), and downregulation of p21 (CIP1/WAF1) (*p* < 0.05) in H1975GR compared to age matched parent H1975P cells. (**B**) Representative Western blot images showing alterations in PI3K/mTOR signaling between H1975P and H1975GR. H1975GR show upregulated p-mTOR (*p* < 0.01), unaltered mTOR and upregulated p-AKT (*p* < 0.01). H1975GR show hyperphosphorylated S6 (*p* < 0.05), in contrast to decreased total 4EBP1 (*p* = 0.0701) and hypo phosphorylated 4EBP1 (Thr37/46) (*p* < 0.01). (**C**) p-4EBP1 (Thr37/46) showed a similar downregulation across all GDC-0980 resistant NSCLC cell line models and p-eIF4B (Ser406) was downregulated in H460GR and A549GR cells compared to the corresponding parent cell lines. (**D**) Conditioned media from H1975GR and H1975P cells were assayed using a magnetic bead based immunoassay (MILLIPLEX MAP), H1975GR secreted higher MCP-1 and TNF-α (both *p* < 0.05), and decreased RANTES (*p* < 0.001) compared to H1975P. * *p* < 0.05, *** *p* < 0.001, paired student *t*-test, *n* ≥ 3. Original Western blots for Figure 6A–C in Figures S8–S10.

The normalized relative abundance of PIM1L, PIM2, PIM3 and c-Myc was significantly higher in the H1975GR cells compared to H1975P, while p21 was significantly lower in the H1975GR by Western blot (Figure 6A). As previously published [12], H1975GR cells demonstrated increased Akt phosphorylation (Ser473) (Figure 6B). Unlike our gene expression data, there was only a modest increase in mTOR protein levels in the H1975GR (Figure 6B). Phosphorylated mTOR (Ser2448) was largely upregulated in the H1975GR cells compared to the parent control (Figure 6B). Ribosomal protein S6 phosphorylation was increased in H1975GR cells while eIF4E-binding protein 1 (4EBP1) was decreased and hypo phosphorylated (Figure 6B). A screen of our panel of the PI3K/mTOR inhibitor resistant cells lines demonstrated a common decrease in p-4EBP1 (Thr37/46) across the panel and p-eIF4B (Ser406) inhibited in H460GR and A549GR cells compared to the age matched parent cell line (Figure 6C).

Cell culture conditioned medium from H1975GR and H1975P cells (*n* = 3) were screened using a MILLIPLEX MAP Magnetic Bead multiplex array panel assaying 10 analytes. INF-γ, IL-12p70, IL-1α and IL-1β were below detectable levels of the ELISA and two were unaltered (IL-6 and IL-8) MCP-1, RANTES and TNF-α secreted proteins levels mirrored the same trend as observed in the IL-6/STAT3 gene array, where H1975GR had increased secretion of MCP-1 (*p* < 0.05) and TNF-α (*p* < 0.05) and decreased secretion of RANTES (*p* < 0.001) (Figure 6D).

### 3.5. Testing the Efficacy of IBL-301 in H1975GR and H1975P Cell Lines

**Figure 7:**
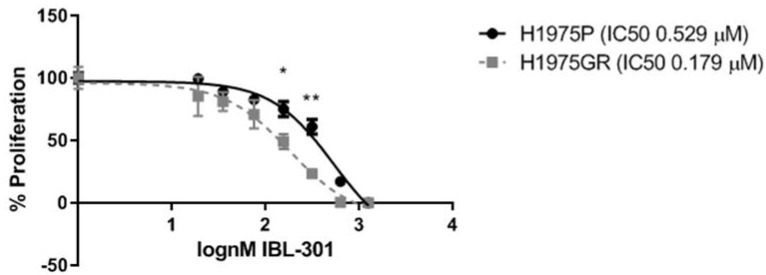
Dose response curves of IBL-301 in H1975GR and matched parent control cell line H1975P. The dose response effect of IBL-301 (0.0195–1.25 μM) on proliferation over 72 h was measured by BrdU assay in the PI3K/mTOR inhibitor resistant cell line (H1975GR) and the age matched parent control cells (H1975P) (*n* = 3). The calculated IC50 values were 0.529 µM and 0.179 µM for the H1975GR and H1975P respectively, indicating that H1975GR cells are more sensitive to the triple targeted PI3K/mTOR/PIM inhibitor (* *p* < 0.05, ** *p* < 0.01, two-way paired *t*-test was performed to compare the values between H1975P and H1975GR cells at each concentration).

H1975GR and H1975P were treated with increasing concentrations (0.0195–1.25 μM) of IBL-301 and inhibition of cell proliferation was measured by the BrdU assay. H1975GR cells were found to be more sensitive to IBL-301 treatment compared to H1975P cells (IC50s 0.179 μM vs. IC50 0.529 μM) (*p* = 0.0047) (Figure 7). This data suggests that targeting PIM kinase in addition to the PI3K/mTOR pathway may provide a novel treatment strategy for patients that develop acquired resistance to PI3K/mTOR inhibitors.

### 3.6. Investigation of Expression of a PIM1 and c-Myc Associated microRNA Signature in PI3K/mTOR Inhibitor Resistant H1975GR

**Figure 8:**
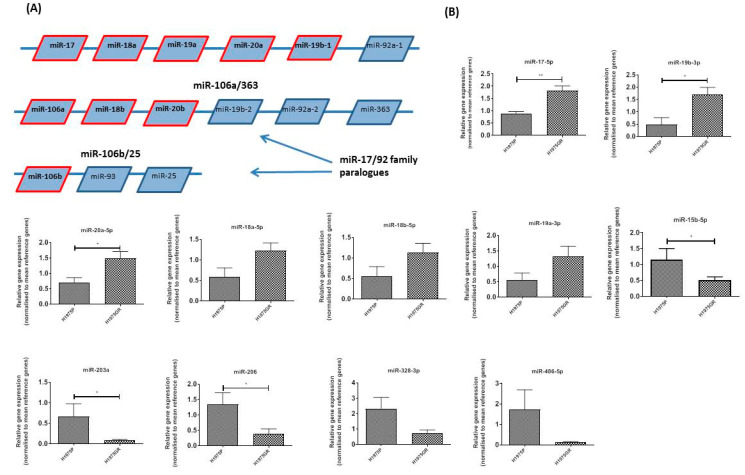
Characterization of PIM1 and MYC associated microRNA signature in PI3K/mTOR inhibitor resistant H1975GR. Small RNA preparations, including miRNAs from both H1975GR and H1975P cells were tested in duplicate wells using a custom miRNA expression array (*n* = 3) containing 19 genes plus endogenous control genes. (**A**) Nine genes within the miR-17/92 cluster and paralogues were included in the custom array and there was a significant upregulation of (**B**) miR-17-5p (*p* < 0.01), miR-19b-3p (*p* < 0.05) and miR-20a-5p (*p* < 0.05) in the H1975GR cells compared to H1975P. There was a similar upregulation for a number of miRNAs in this cluster; miR-18a-5p (*p* = 0.0876), miR-18b-5p (*p* = 0.1467) and miR-19a-3p (*p* = 0.1230) in H1975GR. There was a downregulation of miR15b-5p (*p* < 0.05), miR-203a, miR206 (*p* < 0.05), miR328-3p and miR486-5p (both *p* = 0.1068) in H1975GR compared to age matched control cells, H1975P. * *p* < 0.05, ** *p* < 0.01, paired student *t*-test, *n* = 3.

Small RNA preparations, including miRNAs were extracted from both H1975GR and H1975P age matched parent control cells and analyzed using a custom miRNA expression array panel (*n* = 3) consisting of reported and predicted effectors or regulators of PIM kinase and c-Myc expression. Our miRNA panel contained a subset of nine oncomir-1 and paralogue genes (Figure 8A). There was a significant upregulation of miR-17-5p (*p* < 0.01), miR-20a-5p (*p* < 0.05) and miR-19b-3p (*p* < 0.05) (Figure 8B) in the H1975GR cells compared to H1975P. Although not significant, there was a similar trend of enhanced expression in the H1975GR cells noted for a number of other miRNAs in these clusters, namely miR-18a-5p, miR-18b-5p and miR-19a-3p (Figure 8B). In contrast, miR-106a and miR-106b were unchanged between the two cell lines and miR-20b-5p was below the level of detection in our assay.

The remainder of the miRNA custom array contained three members of the miR-15/16 family (miR-15a-5p, miR-16-1 and miR-15b-5p), miR-203, two members of miR-1/206 cluster (miR-1 and miR-206), miR33-5p (miR-33a and miR-33b), miR328 and miR486-5p. miR-15a-5p and miR-16-1 were unaltered between H1975GR and H1975P, however miR-15b-5p (Figure 8B) was downregulated in H1975GR (*p* < 0.05). miR-203a was downregulated in H1975GR (*p* < 0.05) (Figure 8B). While miR-1 was undetected in either cell line, the other miR-1 family member, miR-206, was downregulated in H1975GR (*p* < 0.05) (Figure 8B). Both miR-33a and miR-33b were unaltered between H1975P and H1975R, miR-328-3p (Figure 8B) and miR-486-5p (Figure 8B) were decreased in H1975GR compared to the parent control cell line, although statistical significance was not reached.

## 4. Discussion

PIM1 is the most studied of the PIM kinase family and while several early studies have shown that tumor PIM1 has a positive impact on patient outcomes [16,17,18]. A large number of studies link PIM1 activity to a more invasive tumor phenotype and worse prognosis in cancers [19,20,21,22,23]. Thus, the PIM kinase pathway has become an attractive target for cancer therapy with a number of drugs under investigation in the clinic including TP-3654 in advanced solid tumors (NCT03715504) and myelofibrosis (NCT04176198) and PIM447 in myelofibrosis (NCT02370706) as well as a number of drugs at preclinical stages of testing [11,24,25,26]. Here we show that all three PIM kinases are expressed in HCC827, H460 and H1975 cell lines and PIM1 positive NSCLC patients had a worse median survival than those that were PIM1 negative (*p* = 0.056).

PIM kinase signaling has been shown to occur in parallel to PI3K/Akt/mTOR signaling with overlapping substrates (e.g., BAD, p21 and p27 among others) [27,28,29,30], suggesting a synergism between these pathways that could enhance resistance to anti-PI3K/Akt/mTOR therapies. Here, for the first time we investigated the efficacy of a novel preclinical PIM/PI3K/mTOR inhibitor IBL-301 in NSCLC. *K-RAS* mutant Calu-6 and *EGFR* (*L858R* and *T790M*)/*PIK3CA* mutant cell lines were both sensitive to IBL-301 in the nanomolar range (IC50 0.3 and 0.9 nM respectively). At a molecular level, although requiring further evaluation with additional replicates, initial results indicate that the dual targeting of PI3K/mTOR or the triple targeting of PI3K/mTOR/PIM kinase are on the whole inhibiting the activation of similar pathways, e.g., Akt and downstream effectors 4EBP1 (Thr37/46) and eIF4B (Ser406). Interestingly both PI3K-mTOR and PI3K/mTOR/PIM kinase inhibition resulted in an initial increase in PIM1S at 2 and 6 h time-points with IBL-301 resulting in the most significant increase. It has been shown previously that small-molecule PIM inhibitors lead to an increase in PIM protein levels [31,32]. However, IBL-301 was more effective at inhibiting this increase in PIM1S at 24 h and it also inhibited PIM1L. Triple-targeting IBL-301 arguably showed a unique ability to downregulate c-Myc. Given the role of PIM kinase in stabilizing c-Myc [33,34,35,36], it is not surprising that c-Myc levels are decreased by an inhibitor with this unique PIM kinase targeting arm. IBL-301 inhibited activation of several proliferative pathways including the MAP kinase pathway proteins ERK and p38, PI3K-Akt pathway and JAK/STAT pathway in NSCLC tumor explants. Significant cross-talk between these pathways provides an escape mechanism for cancer cells, a characteristic of acquired resistance to targeted therapies. The tissue conditioned media, treated with IBL-301, also had significantly reduced levels of pro-inflammatory chemokine MCP-1 (CCL2), an inducer of EMT [13] and a biomarker of poor prognosis in NSCLC [37]. Further studies are warranted to elucidate whether IBL-301 can also modulate the immune response and activate cytotoxic T lymphocytes (CTLs) in NSCLC.

Next we examined our PI3K-mTOR inhibitor resistant cell lines and PIM1L was elevated in H1975GR and A549GR cells compared to age matched parental cells. PIM1 expression was not elevated in the H460GR resistant cells compared to the H460P parent cells and this may be due to the presence of other resistant clones that do not have activated PIM kinase but are driven by a different mechanism of resistance that are also present. Further in-depth analysis of the IL-6/STAT-3 signaling pathway using the gene profiler array showed upregulation of PIM1 and co-regulator MYC in the H1975GR cells. Overexpression was robustly validated at both mRNA and protein levels. PIM1 has two protein isoforms PIM1S (34 kDa) and PIM1L (44 kDa) [38], with the 44 kDa isoform being predominantly overexpressed in H1975GR cells. Increased PIM1L is thought to have a role in drug resistance in prostate cancer [3,39], which is in line with our novel findings in NSCLC. In addition, PIM2 and 3 were also found to be overexpressed in H1975GR cells. PIM kinases alone are only weakly oncogenic, however when co-expressed with c-Myc they can phosphorylate/stabilize it, enhancing c-Myc-driven tumorigenicity [33].

The phosphorylation of AKT and mTOR were upregulated in H1975GR cells compared to the age matched parent control. The upregulation of mTOR activity in H1975GR cells may be due to overexpression of PIM1 and its overlapping role with Akt to regulate the mTOR inhibitory protein PRAS40 via phosphorylation [40]. Unexpectedly the downstream effector molecule of mTOR, 4E-BP1 (Thr37/46) was hypo phosphorylated compared to the age matched control cells. During mTOR signaling the translation suppressor protein 4E-BP1 is normally phosphorylated, causing it to dissociate from the eukaryotic initiating factor eIF4E, which is then free to be phosphorylated/activated and to complex with other eIFs to initiate cap-dependent translation. 4EBP1 is also a known substrate of PIM1 kinase activity [24,41] however it does not appear to be altered by the amplified PIM1 expression in the H1975GR cells. This is further supported by the observation of a common decrease in phosphorylation of 4EBP1 (Thr37/46) across the three PI3K/mTOR inhibitor resistant cell lines models and phosphorylation of eIF4B (Ser406) in the H460GR and A549GR cells. 4EBP1 and ribosomal protein S6 are two of the best characterized downstream effectors of PI3K/Akt/mTOR signaling. In contrast to 4EBP1, ribosomal protein S6 had increased activity in the H1975GR cells as indicated by increased S6 phosphorylation compared to H1975P. S6 forms part of the 40S ribosomal subunit which together with eiF3 are essential for both cap-dependent and cap independent translation. Others have provided evidence for PI3K/mTOR inhibitor resistance induced cell survival mechanisms involving enhanced cap-independent translation [42]. It is also reported that endogenous mRNAs coding PIM1 and MYC can be translated in a cap-independent manner when cap-dependent translation is compromised, e.g., during drug induced cellular stress [43,44]. Furthermore, PIM1 may be essential for IRES-mediated cap independent translation of elements including c-Myc in drug resistance responses to Akt inhibitors [5] and MET inhibitors [6]. Thus, the observed overexpression of PIM1/2/3 and c-Myc in our PI3K/mTOR inhibitor resistant H1975GR cells may occur through a cap independent mechanism.

Overexpression of BCL2 in tandem with MYC overexpression in H1975GR cells is of critical importance in our drug resistance model. Paradoxically while MYC is a potent oncogene that can cause continuous cell proliferation, it is also capable of inducing massive apoptosis involving tumor suppressors ARF and p53 [45]. Concomitant Bcl-2 expression with c-Myc is required to block c-Myc induced apoptosis and to tip the balance in favor of c-Myc-induced transformation and survival. In contrast to c-Myc, we saw inverse mRNA levels of CDKN1A/p21. Overexpression of c-Myc has been shown to downregulate a number of growth/cell cycle arrest genes including p15, p27 and p21 and c-Myc has been shown to repress p21 gene expression by preventing the interaction of key transcription factors with p21 promoter region, leading to cell cycle progression [46,47]. Additionally, p21 is a substrate of PIM1, with its phosphorylation leading to protein destabilization and degradation [24] which is in line with the decreased p21 protein levels observed in the H1975GR cells. A study by Liu et al. in NSCLC cells demonstrated that downregulation of PIM2 resulted in upregulation of p21, irrespective of the p53 status of the cells [48]. We observed a downregulation of tumor suppressor SOCS1 (suppressor of cytokine signaling 1) gene expression in the H1975GR cells. Downregulated SOCS1 results in increased cell proliferation and decreased apoptosis which is consistent with our drug resistant cell line model [49]. The H1975GR cells showed a massive increase in expression of TNF mRNA and more modest increase in secreted levels compared to the age-matched control cells. However, the likely outcome of this is unknown given the fact that tumor or microenvironment derived TNF-α can have paradoxical tumor promoting and inhibiting roles. While high levels of tumor derived TNF-α would certainly impede tumor growth and lead to necrosis and increase immune activation and attack, continuous low levels of intra-tumor TNF-α can stimulate myeloid cell infiltration (e.g., TAMS, tumor associated macrophages), increase vessel innervation and enhance EMT and/or dedifferentiated stem-like phenotype [50]. Finally, a reciprocal gene and protein expression of CCL2 (MCP-1) and CLL5 (RANTES) was observed in the H1975GR. The decreased levels of RANTES in the H1975GR may be tumor-protective in the scenario of drug resistant lung cancer, given that high RANTES is sometimes associated with an improved patient survival [51], most likely as a result of enhanced cytotoxic T-cell recruitment and tumor cell killing, although further research is required to define the relationship between these correlations [52]. The increased levels of MCP-1 observed in the H1975GR will most likely have a pro-tumorigenic effect, given that pharmacological inhibition of MCP-1 reduces tumor growth and augments macrophage phenotype and activates cytotoxic T-cells [53,54]. Additionally, MCP-1 appears to be involved in chemo-resistance mechanisms in lung cancer [55].

In light of our novel finding that all three isoforms of PIM kinase were upregulated, we hypothesized that the H1975GR cells switched to this parallel, compensatory pro-tumorigenic pathway, in order to overcome the inhibitory effect of the PI3K/mTOR antagonist and to sustain growth. Interestingly, H1975GR cells were more sensitive to treatment with the novel IBL-301 than the age matched H1975P cells indicating a reliance on PIM kinase signaling. Further studies are required to define the precise mechanism by which PIM kinases promote drug resistance in response to PI3K-mTOR inhibition in our NSCLC models. Previous studies have shown that PIM is able to reactivate signaling cascades downstream of Akt to promote translation and suppress apoptosis and PIM1 increased expression of RTKs in prostate cancer patients in a cap-independent manner via control of internal ribosome entry [5]. Song et al. showed that PIM kinase can induce tumor cell resistance to inhibitors of PI3K/AKT signaling by increasing nuclear factor E2-related factor 2 (NRF2) levels and stimulating the production of ROS scavengers [56]. We hypothesize that PIM kinases may also corroborate with metastasis associated in colon cancer 1 (MACC1), a known driver of drug resistance, to reactivate the PI3K/Akt pathway [57,58]. MACC1 expression has been shown to correlate with PIM3 expression and overexpression of PIM3 in ovary cancer cells increased MACC1 mRNA and protein expression [59]. We and others have suggested the synergism of targeting PI3K/Akt/mTOR and PIM kinase to combat resistance to current PI3K/Akt/mTOR inhibitors [4,5,11,24,25,30] and our findings further support this rationale.

To identify a miRNA signature that could be used to monitor therapeutic resistance we screened 19 miRNAs with a reported role (as either regulators or effectors) in c-Myc and PIM kinase tumor activity or for their predicted regulatory role determined by TargetScan [60]. The miR-17/92 cluster or “oncomir-1” and its two human paralogue clusters miR-106a-363 and miR-106b-25 consist of 15 miRNAs that can be transcriptionally regulated by both c-Myc and PIM1 kinase [61,62,63,64]. Here, we report a significant upregulation of miR-17-1, miR-20a-5p, miR-19b-3p and a similar trend for several other members of this family in the H1975GR cells compared to H1975P. This is in line with the significant upregulation of both of the established miR-17/92 regulators c-Myc and PIM1 observed in these cells. Along with c-Myc, miR-17-1 and miR-20a are known to target p21 (CDKN1A) [65,66]. Thus c-Myc and PIM1 together with the miR-17/92 cluster may have a role in the development of resistance to PI3K/mTOR inhibitor Apitolisib, at least in part through a modulation of p21. In this study we report a decrease in miR-15-5p, which is part of the miR-15/16 family known to play very important roles in regulating cell proliferation and apoptosis, in addition to having conserved target sites on PIM1 mRNA and a reported regulatory role [67,68,69]. The miR-15b/16-2 is also transcriptionally regulated by c-Myc [70]. The tumor suppressor miR-203a is significantly decreased in the H1975GR cells and has previously been identified as a regulator of MYC transcription [71]. The miR-1/206 cluster, miR33-5p, miR328 and miR486-5p are all reported in the literature as regulators of untranslated PIM1 or other PIM kinases [2,72,73,74,75]. While miR-1 was undetected in either cell line, another miR-1 family member, miR-206, was downregulated in H1975GR. Both miR-33a and miR-33b were unaltered between H1975P and H1975P, while miR-328-3p and miR-486-5p showed a decreasing trend in expression in the H1975GR cells. Thus, we are seeing a reciprocal regulation of c-Myc/PIM1 and a number of bona fide tumor suppressor miRNAs.

## 5. Conclusions

The present study demonstrates that all three PIM kinase are expressed in NSCLC. Elevated PIM1 expression correlates to a poor prognosis and PIM kinases are activated in response to PI3K-mTOR inhibition in NSCLC. IBL-301 inhibited the PI3K-Akt and JAK/STAT pathways in vitro and in NSCLC tumor tissue explants and also inhibited secreted pro-inflammatory cytokine MCP-1 and warrants further preclinical and clinical investigation. These results, together with previous data in other cancer types, highlight the potential for dual targeting of both PIM kinase and PI3K-mTOR pathways as a promising strategy for the future treatment of patients with PIM kinase and/or PI3K-mTOR activated NSCLC.

## Data Availability

All the data generated in this study is contained within the article or supplementary material.

## Supplementary Materials

The following are available online at https://www.mdpi.com/article/10.3390/cancers13092139/s1, Figure S1: (A) The precise synthetic structure of triple PI3K, mTOR and PIM1/2/3 kinase inhibitor IBL-301 and (B) IC50 of 2.5, 134, 18.2, 13.6 and 3.17 nM respectively, Figure S2: IL-6/STAT3 gene array expression profile of GDC-0980 resistant H1975 (H1975GR) versus parent H1975 (H1975P). A clustergram of the 84-gene array provides a heat map of gene clusters differentially co-regulated between H1975GP and H1975GR, Figure S3: Original Western blots for Figure 1A, Figure S4: Original Western blots for Figure 3B, Figure S5: Original Western blots for Figure 3C, Figure S6: Original Western blots for Figure 3D, Figure S7: Original Western blots for Figure 5A, Figure S8: Original Western blots for Figure 6A, Figure S9: Original Western blots for Figure 6B, Figure S10: Original Western blots for Figure 6C, Table S1: NSCLC Patient Clinicopathological Characteristics, Table S2: Patient characteristics for ex-plant tumor tissues treated with BEZ235 or IBL-301, Table S3: Forward and Reverse Primer Sequences used for mRNA expression validations by qPCR, Table S4: Name of miRNAs included in array panel and their corresponding Exiqon probe catalogue names.
