# Supplementary material for: Co-Targeting PIM Kinase and PI3K/mTOR in NSCLC
Source: Cancers (Basel). 2021 Apr 29;13(9):2139. doi: 10.3390/cancers13092139 (PMC8125027; doi:10.3390/cancers13092139)
Supplement: Supplementary file 1 [file cancers-13-02139-s001.zip › cancers-1122337-supp-final.pdf]

Supplementary Materials: Co-Targeting PIM Kinase and PI3K/mTOR in NSCLC

Gillian Moore, Clara Lightner, Samira Elbai, Lauren Brady, Siobhan Nicholson, Ronan Ryan, Katie E. O'Sullivan, Kenneth J. O'Byrne, Carmen Blanco-Apiricio, Sinead Cuffe, Michael O'Neill, Susan Heavey, Stephen P. Finn and Kathy Gately

(A)

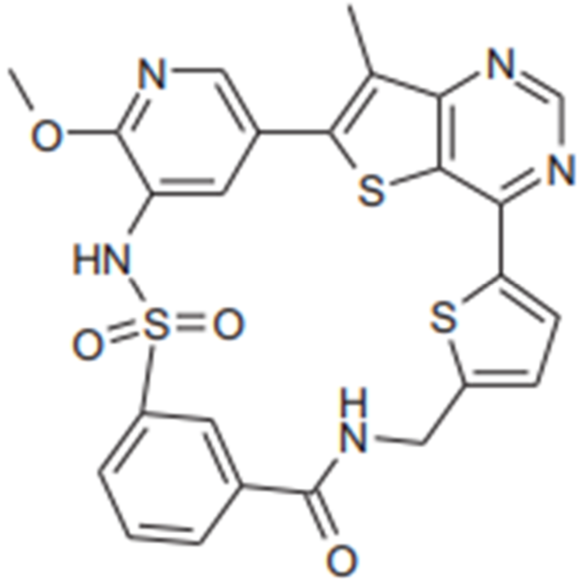

(B)

IC50 (nM)					
	PI3Kα	mTOR	PIM1	PIM2	PIM3
IBL-301	2.5	134	18.2	13.6	3.17

Figure S1. (**A**) The precise synthetic structure of triple PI3K, mTOR and PIM1/2/3 kinase inhibitor IBL-301 and (**B**) IC50 of 2.5, 134, 18.2, 13.6 and 3.17 nM respectively.

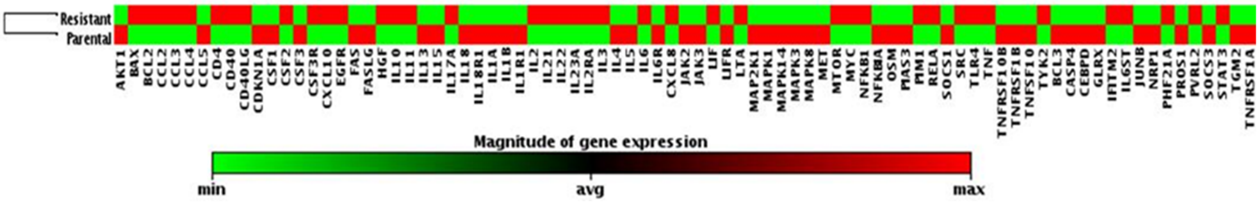

Figure S2. IL-6/STAT3 gene array expression profile of GDC-0980 resistant H1975 (H1975GR) versus parent H1975 (H1975P). A clustergram of the 84-gene array provides a heat map of gene clusters differentially co-regulated between H1975GP and H1975GR.

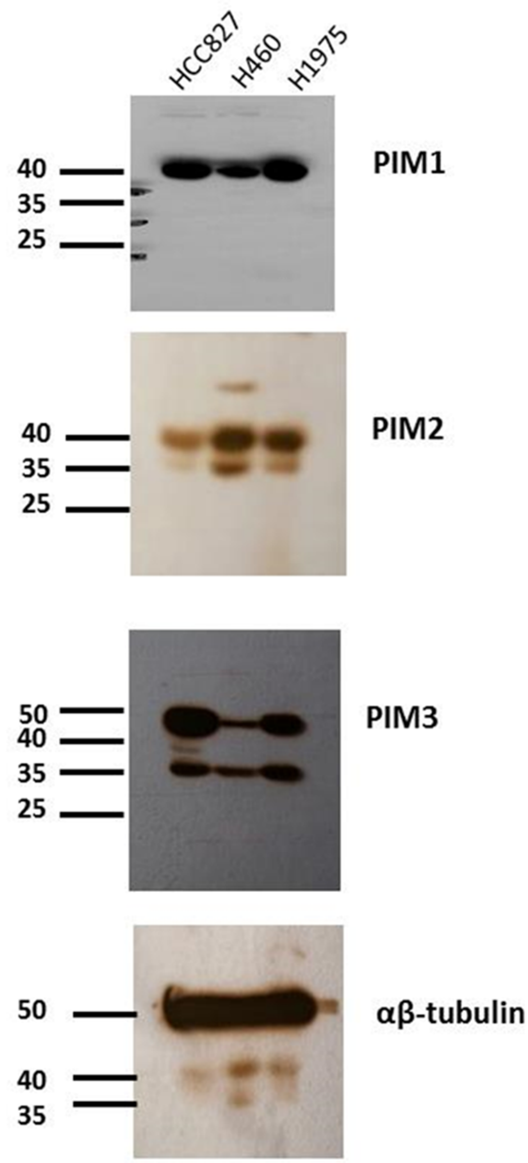

Figure S3. Original Western blots for Figure 1A.

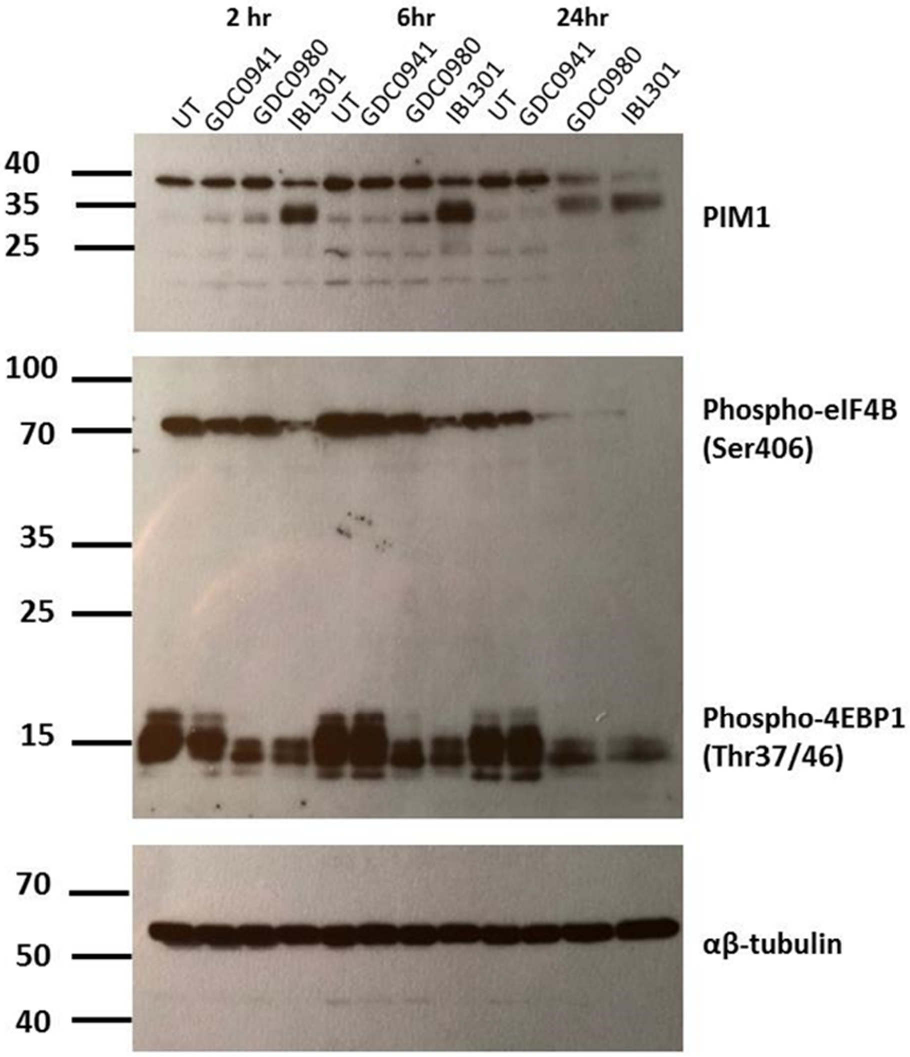

Figure S4. Original Western blots for Figure 3B.

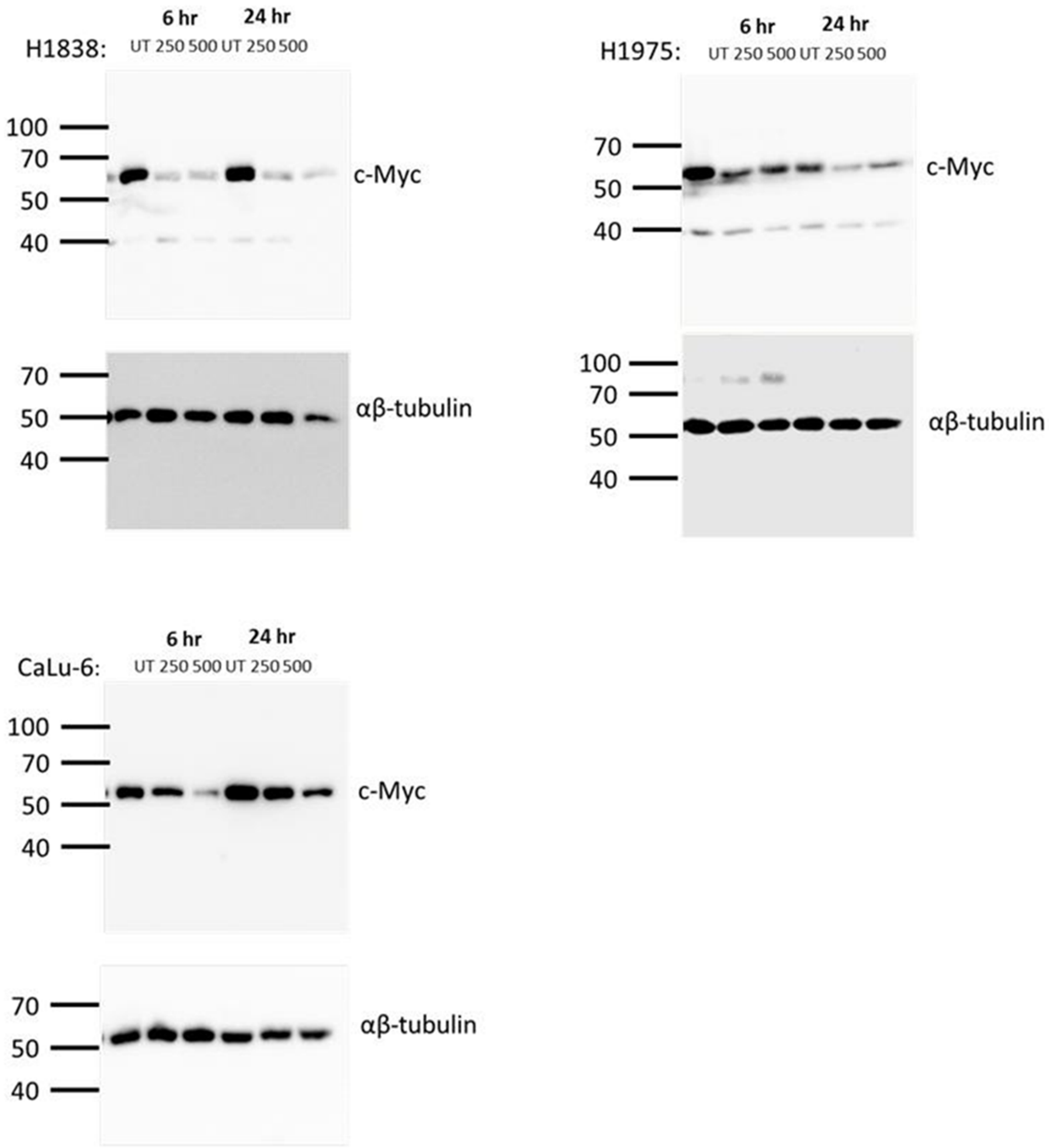

Figure S5. Original Western blots for Figure 3C.

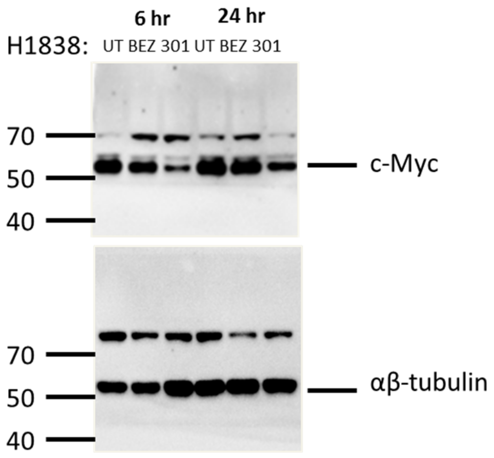

Figure S6. Original Western blots for Figure 3D.

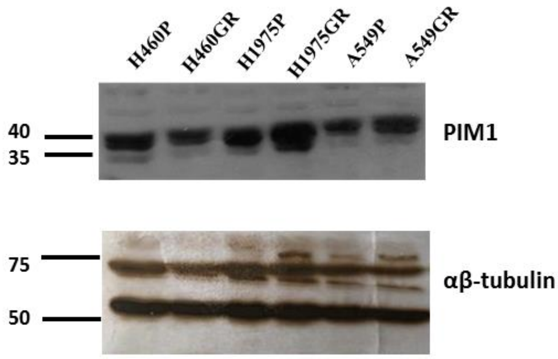

Figure S7. Original Western blots for Figure 5A.

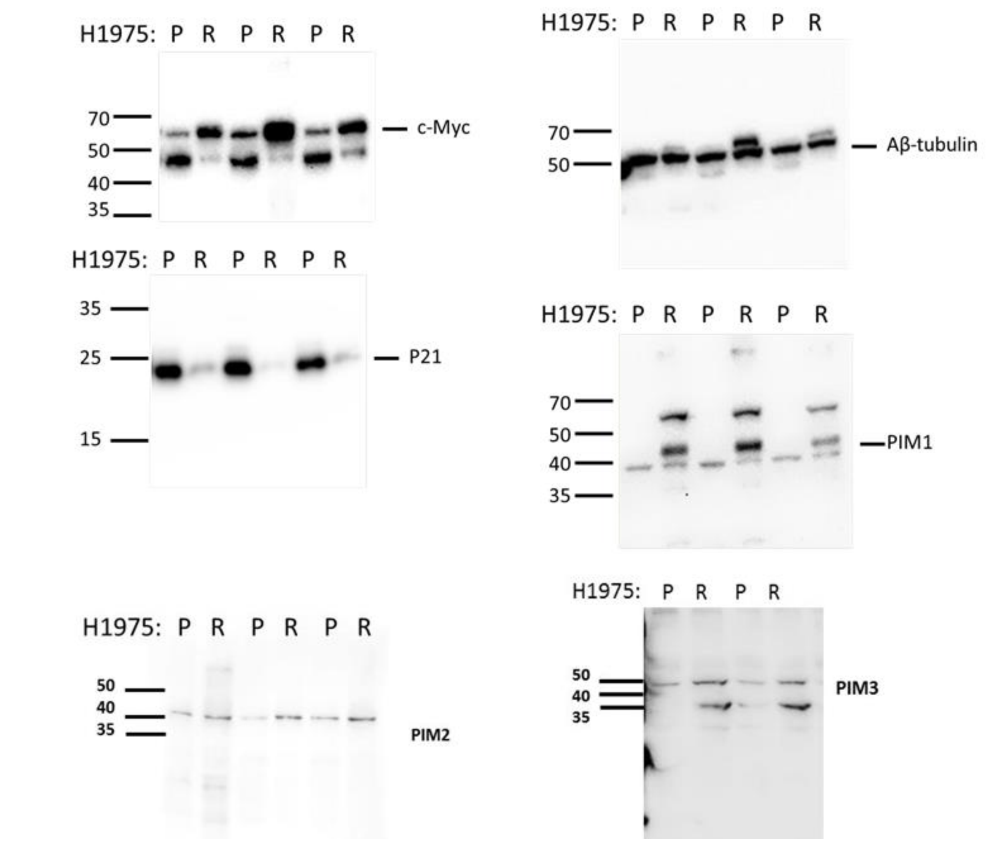

Figure S8. Original Western blots for Figure 6A.

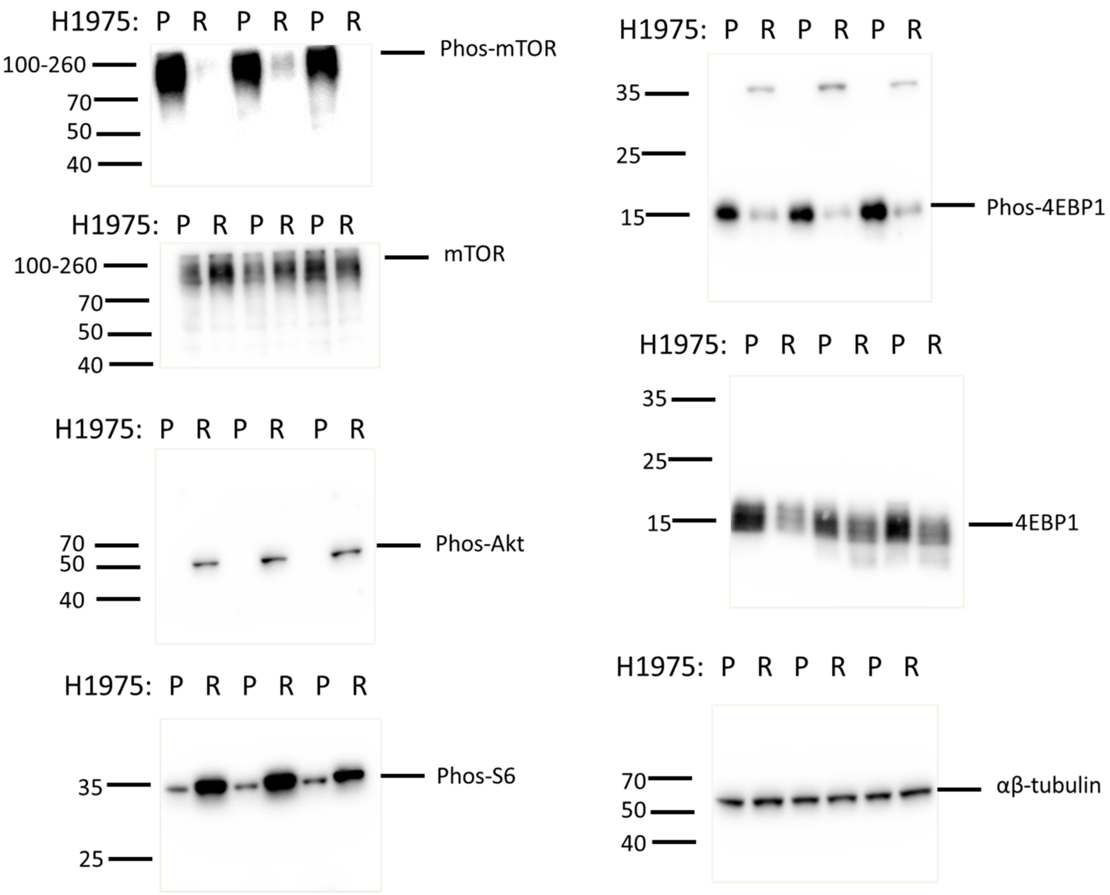

Figure S9. Original Western blots for Figure 6B.

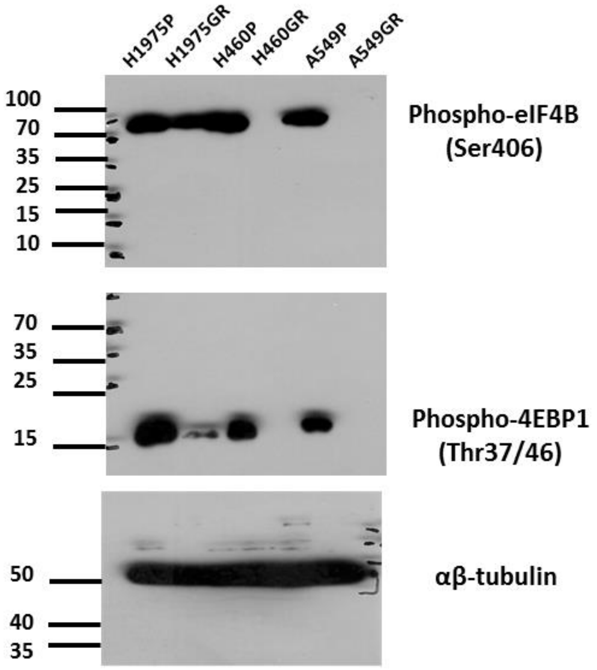

Figure S10. Original Western blots for Figure 6C.

Table S1. NSCLC Patient Clinicopathological Characteristics.

**Age (mean ±S.D.)**	64 (±9.1)
**Sex (%):**	
Male	85 (63.4%)
Female	49 (36.6%)
**Smoking Status (%)**	
Current	65 (48.5%)
Ex-Smoker	47 (35.1%)
Non-Smoker	22 (16.4%)
**Histology (%)**	
Adenocarcinoma	60 (44.8%)
Squamous Cell Carcinoma	65 (48.5%)
Other	9 (6.7%)
**Grade (%)**	
1	12 (9.0%)
2	80 (59.7%)
3	42 (31.3%)
**Tumor size (cm) (mean ±S.D.)**	4.87 (±2.61)
**T-stage (%)**	
IA-IB	71 (53.0%)
IIA-IIB	35 (26.1%)
IIIA-IIIB	27 (20.2%)
IV	1 (0.7%)

Nodal Involvement (%)	
Yes	54 (40.3%)
No	80 (59.7%)

Table S2. Patient characteristics for ex-plant tumor tissues treated with BEZ235 or IBL-301.

Histology	Age	Gender	Stage	Sample Type Tested
Adenocarcinoma	73	Male	IIIA	Tissue & TCM
Adenocarcinoma	67	Male	IIIA	Tissue & TCM
Squamous Cell	66	Male	IIIA	Tissue & TCM
Squamous Cell	76	Female	IA	TCM
Adenocarcinoma	71	Male	IIIA	TCM
Adenocarcinoma	78	Female	IB	TCM
Squamous Cell	71	Female	IIIA	TCM
Adenocarcinoma	70	Male	IIIA	TCM
Adenocarcinoma	77	Female	IIIA	TCM

Resected lung tumor samples were dissected, cultured and treated with DMSO vehicle control (UT), 250nM BEZ235 or 250nM IBL-301. After 72 hr treatment tissue and tissue culture media (TCM) were stored at -80oC. Patient histology, age, gender and tumor staging AJCC (7th Ed) are summarized in the Table.

Table S3. Forward and Reverse Primer Sequences used for mRNA expression validations by qPCR.

Gene Name	Primer Sequence:
MYC	FW: CTTCTCTGAAAGGCTCTCCTTG
	REV: GTCGAGGTCATAGTTCCTGTTG
PIM1	FW: GAAACAGGTTGGGATGGGATAG
	REV: CACAGAAGGCACTCAGAAAGA
MTOR	FW: GGTCGTGGAGAACATGGATTAG
	REV: ACCAGTGAGGTCTTGGGATA
SOCS1	FW: CTTCTGTAGGATGGTAGCACAC
	REV: AGAACCTTCCTCCTCTTCCT
CDKN1A1	FW: CGGAACAAGGAGTCAGACATT
	REV: AGTGCCAGGAAAGACAACTAC
BCL2	FW: GGCCAGGGTCAGAGTTAAATAG
	REV: GGAGGTTCTCAGATGTTCTTCTC
TNF	FW: GATCCCTGACATCTGGAATCTG
	REV: GAAACATCTGGAGAGAGGAAGG
CD40	FW: CATCCAGTCTCCCAACTTGTAT
	REV: CGGAAGGTCTGGTGGATATTAC
B2M	FW: CCAGCGTACTCCAAAGATTCA
	REV: TGGATGAAACCCAGACACATAG
RPLP0	FW: ATGTTGCCAGTGTCTGTCTG
	REV: AGCAAGTGGGAAGGTGTAATC

Table S4. Name of miRNAs included in array panel and their corresponding Exiqon probe catalogue names.

miR Name	Exiqon Probe Name:
hsa-miR-17-5p	204771
hsa-miR-20a-5p	204292
hsa-miR-20b-5p	204755
hsa-miR-18a-5p	204207
hsa-miR-19a-3p	205862

hsa-miR-19b-3p	204450
hsa-miR-18b-5p	204084
hsa-miR-15a-5p	204066
hsa-miR-15b-5p	204243
hsa-miR-16-5p	205702
hsa-miR-486-5p	204001
hsa-miR-1	204344
hsa-miR-206	206073
hsa-miR-33a-5p	205690
hsa-miR-33b-5p	205860
hsa-miR-328-3p	204364
hsa-miR-203a	205914
hsa-miR-106a-5p	204563
hsa-miR-106b-5p	205884
hsa-miR-103a-3p	204063
hsa-miR-423-5p	205624
hsa-miR-191-5p	204306
